# Impact of serous macular detachment on visual recovery in retinal vein occlusion treatment

**DOI:** 10.5935/0004-2749.20220088

**Published:** 2025-02-11

**Authors:** Ilknur Tuncer Firat, Nihat Polat, Murat Firat

**Affiliations:** 1 Department of Ophthalmology, Inonu University School of Medicine, Malatya, Turkey; 2 Department of Ophthalmology, Malatya Training and Research Hospital, Malatya, Turkey

**Keywords:** Retinal vein occlusion, Macular edema, Macular detachment, Intravitreal injections, Oclusão da veia retiniana, Edema macular, Descolamento macular, Injeções intravítreas

## Abstract

**Purpose:**

The aim of this study was to evaluate the effect of serous macular detachment observed during retinal vein occlusion on treatment results.

**Methods:**

A total of 117 eyes from 115 patients who had been treated with intravitreal injections for macular edema secondary to retinal vein occlusion were retrospectively reviewed. Visual acuity, optical coherence tomography, and fundus fluorescein angiography findings were evaluated according to the status of serous macular detachment.

**Results:**

In the branch retinal vein occlusion group, a statistically significant increase was detected in the mean visual acuity compared to the baseline value at each visit in the absence of serous macular detachment, whereas the increase in the mean visual acuity was significant only at the 3- and 6-month visits in the presence of serous macular detachment. In the central retinal vein occlusion group, there was an increase in the mean visual acuity compared to the baseline value at every visit in the absence of serous macular detachment, whereas the mean visual acuity decreased compared to the baseline value at every visit except at the 3-month visit in the presence of serous macular detachment. The ellipsoid zone defect was more prominent in the presence of serous macular detachment in eyes with branch retinal vein occlusion, whereas there was no significant difference in the ellipsoid zone in the absence or presence of serous macular detachment in eyes with central retinal vein occlusion.

**Conclusions:**

In the group with macular edema due to retinal vein occlusion, the initial mean visual acuity increase observed in the first year was maintained in cases without serous macular detachment but not in those with serous macular detachment. Serous macular detachment could be a negative factor in eyes with retinal vein occlusion.

## Abstract (pt)

**Objetivo:**

Avaliar o efeito do descolamento macular seroso observado durante oclusões de veias retinianas nos resultados do tratamento.

**Métodos:**

Um total de 117 olhos de 115 pacientes que foram tratados com injeções intravítreas para edema macular secundário à oclusão de veia retiniana foram revistos retrospectivamente. A acuidade visual, tomografia de coerência óptica e os resultados da angiofluoresceinografia foram avaliados de acordo com a presença ou ausência de descolamento macular seroso.

**Resultados:**

No grupo com oclusão de um ramo da veia retiniana, foi detectado um aumento estatisticamente significativo na acuidade visual média em comparação com o valor inicial em cada consulta de acompanhamento do descolamento macular seroso, enquanto que o aumento na acuidade visual média só foi significativo nas consultas aos 3 e 6 meses na presença de descolamento macular seroso. No grupo com oclusão da veia central da retina, houve um aumento na acuidade visual média em comparação com a acuidade inicial em cada consulta na ausência de descolamento macular seroso, enquanto a acuidade visual média diminuiu em comparação com a acuidade inicial em todas as consultas, exceto na consulta aos 3 meses. O defeito da zona elipsoide era mais proeminente na presença de descolamento macular seroso nos olhos com oclusão de um ramo da veia retiniana, enquanto que não havia diferença significativa na zona elipsoide com a presença ou ausência de descolamento macular seroso em olhos com oclusão central da veia retiniana.

**Conclusões:**

No grupo com edema macular devido à oclusão de veias retinianas, o aumento médio inicial da acuidade visual observado no primeiro ano foi mantido nos casos sem descolamento macular seroso, mas não naqueles com presença de descolamento macular seroso. O descolamento macular seroso pode ser um fator negativo em olhos com oclusão de veias retinianas.

## INTRODUCTION

Retinal vein obstruction (RVO) is the most common retinal vascular disorder that develops after diabetic retinopathy^(1)^. One of the basic reasons for vision loss in RVO is macular edema^(2)^. Some eyes with macular edema in RVO have demonstrated a less pronounced response to the same treatments than other eyes^(3-6)^. This finding has led to the investigation of various prognostic factors. Previously detected prognostic factors for branch retinal vein obstruction (BRVO) include initial visual acuity (VA), age, BRVO duration, macular ischemia, cystic areas of >600 µm in the fovea, and defects in the ellipsoid zone and the external limiting membrane (ELM)^(4-7)^. Some studies have reported that serous macular detachment (SMD) secondary to RVO has a positive or negative effect on the results, whereas other studies have found no effect^(4,6,8)^. The aim of this study was to determine the effect of SMD on the treatment and prognosis of macular edema due to RVO in a large patient group.

## METHODS

We retrospectively reviewed the charts of patients who had been diagnosed with macular edema due to RVO at the İnönü University Faculty of Medicine Turgut Özal Medical Center’s Ophthalmology Clinic between January 2011 and July 2017 and subsequently treated with an intravitreal (IV) sustained-release dexamethasone implant (Ozurdex; Allergan, Inc., Irvine, CA, US), bevacizumab (Avastin; Genentech Inc., South San Francisco, CA, US), ranibizumab (Lucentis; Genentech Inc., South San Francisco, CA, US), or aflibercept (Eylea, Regeneron Inc, NY, US). This study was conducted according to the principles of the Declaration of Helsinki and patient treatment. Consent from the Malatya İnönü University Ethics Committee was obtained before conducting the study (İnönü University Scientific Research and Publication Ethics Committee - Health Sciences Noninterventional Clinical Studies Ethics Committee - Decision No: 2018/9-11).

Patients who participated in the first visit and at least one of the 3-, 6-, 9- and 12-month visits with an optical coherence tomography (OCT) image suitable for evaluation were included in the study. The last visit the patient could attend was accepted as the final visit.

We excluded patients treated at an external center. Those with the following conditions were also excluded: presence of choroidal neovascularization, diabetic retinopathy, or significant media opacity; vitreomacular disorders at baseline; a history of other disorders that could affect vision such as retinal artery occlusion and ocular inflammation; past vitreoretinal surgery; grid laser treatment to the macula; and poor OCT image quality.

Before the injection and laser treatment, written and verbal informed consent was obtained from all patients. Patients recruited early in the study could not initially receive anti-vascular endothelial growth factor (VEGF) treatment for RVO-related macular edema because of limitations in the Turkish National Health System and were instead treated with IV sustained-release dexamethasone twice a year at most. After the approval of IV anti-VEGF treatment for RVO, patients who had not responded or had demonstrated a poor response to previous IV sustained-release dexamethasone treatment were started on one of the IV anti-VEGF treatments as an additional treatment. After the approval of IV anti-VEGF treatments, patients with RVO receiving treatment for the first time were primarily assigned to one of the anti-VEGF options, but an IV sustained-release dexamethasone implant was placed later in case the treatment response was inadequate. After IV anti-VEGF treatment had been administered monthly for 3 months, or after the first IV sustained-release dexamethasone treatment was administered, the patient was called in for a 3-month follow-up if there was a good response or for monthly treatment if the response was poor.

The criteria for treatment initiation were the presence of a central foveal thickness (CFT) of >250 µm and/ or a significant intraretinal cyst or subretinal fluid due to RVO. The criteria for retreatment and treatment continuation were an increase of >50 µm in CFT since the last visit and/or a marked increase in intraretinal cysts, an inadequate response to previous treatment, and a loss of two or more lines in VA on the ETDRS scale. IV treatment was stopped if there was no response to three consecutive IV injections, and patients were called for a follow-up visit 3-6 months later according to their risk for other complications.

Data regarding age, sex, the presence of diabetes mellitus or hypertension, eye with RVO, RVO type, ischemic status, VA (logMAR), CFT measurement (µm) by OCT (spectral-domain OCT), the presence of SMD, the presence of foveal ellipsoid zone and ELM damage at the final visit, the presence of initial macular ischemia, the type of injection administered, and the number of doses were collected. The results of fundus fluorescein angiography (FFA) were considered to be ischemic if the area was >5 disk areas for BRVO and 10 disk areas for central retinal vein occlusion (CRVO)^(9)^. CFT was defined as the mean thickness within the central, 1-mm-diameter foveal area and was obtained from the OCT thickness map. SMD was defined as the presence of any hyporeflective area between the neurosensorial retina and RPE, together with macular elevation. Any disruption of the ellipsoid zone or ELM at the fovea was accepted as the presence of ellipsoid zone or ELM damage. Patients were evaluated in two groups as BRVO and CRVO according to the type of RVO. They were further divided into two subgroups as SMD absent (SMD (-)) and SMD present (SMD (+)).

### Statistical analysis

The SPSS Windows version 22.0 (SPSS Inc., Chicago, IL, US) software was used for statistical analysis. Results are expressed as mean ± standard deviation (SD). The Kolmogorov-Smirnov test was used to determine whether the quantitative data conformed to a normal distribution. In the dependent groups, a paired *t*-test was used for data with a normal distribution, and the Wilcoxon signed-rank test was used for data without a normal distribution, and the respective tests used in the independent groups were an unpaired *t*-test and the Mann-Whitney U test. Qualitative variables were evaluated using Pearson chi-square or Fisher’s exact tests. The level of statistical significance was set at p<0.05.

## RESULTS

A total of 117 eyes from 115 patients were included in this study, of which 75 eyes (64.1%) had BRVO, and 42 eyes (35.9%) had CRVO. There were 62 (53.0%) SMD (-) eyes and 55 (47.0%) SMD (+) eyes in the entire group. No significant difference was found in the mean age, sex, presence of hypertension or diabetes mellitus, laterality, RVO type, presence of ischemia, initial mean VA, or mean CFT value between the groups when divided according to the SMD status (Table 1). SMD was present in 31 (41.34%) eyes in the BRVO group and in 24 (57.1%) eyes in the CRVO group. There was no difference in sex, presence of hypertension and diabetes mellitus, laterality, presence of ischemia, or the total number of injections according to the SMD status in the BRVO and CRVO groups (p>0.05). Among patients with BRVO, the mean age was 60.63 ± 11.78 years in the SMD (-) group and 59.22 ± 12.12 years in the SMD (+) group (p=0.830). in the CRVO group, the mean ages were 64.78 ± 6.25 and 55.0 ± 15.43 years, respectively (p=0.008).

**Table 1:** Initial patient characteristics according to SMD status

	SMD (-)	SMD (+)	p value
	(n=62 (53.0%))	(n=55 (47.0%))	
Male gender, n (%)	39 (62.9)	29 (52.7)	0.355
Age, years (mean ± SD)	61.84 ± 10.60	57.38 ± 13.70	0.140
Hypertension presence, n (%)	31 (50.0)	24 (43.6)	0.491
Diabetes mellitus presence, n (%)	8 (12.9)	12 (21.8)	0.302
Laterality, n (%) (Right/Left)	32 (51.6) / 30 (48.3)	31 (56.4) / 24 (43.6)	0.607
RVO type, n (%) (CRVO / BRVO)	18 (29.0) / 44 (71.0)	24 (43.6) / 31 (56.4)	0.147
Ischemia presence, n (%)	22 (35.5)	14 (25.5)	0.331
VA, logMAR (mean ± SD)	0.86 ± 0.46	0.87 ± 0.46	0.944
CFT, µm (mean ± SD)	590.11 ± 201.11	589.56 ± 203.02	0.920
BRVO Fol. Up (Months) (mean ± SD)	17.68 ± 11.27	16.58 ± 9.68	0.931
CRVO Fol. Up (Months) (mean ± SD)	19.61 ± 10.13	15.04 ± 11.69	0.073

BRVO= branch retinal vein occlusion; CRVO= central retinal vein occlusion; RVO= retinal vein occlusion; SMD= serous macular detachment; CFT= central foveal thickness; VA= visual acuity; Fol. Up= Follow up time.

The mean number of anti-VEGF injections was 2.37 ± 1.46 in the SMD (-) group and 2.43 ± 1.43 in the SMD (+) group (p=0.828). The mean number of Ozurdex injections was 1.71 ± 0.91 in the SMD (-) group and 1.56 ± 0.97 in the SMD (+) group (p=0.281). The distribution of IV treatments was similar in SMD (-) and SMD (+) cases in both the BRVO and CRVO groups (Table 2).

**Table 2:** Distribution of the treatment modalities of patients according to the RVO type and SMD status

RVO Type	SMD Status	Dexa. imp.	Ranibizumab	Aflibercept	Bevacizumab	Dexa.Imp. + Ranibizumab	Dexa. Imp. + Aflibecept	Dexa. Imp. + Bevacizumab
BRVO	(-)	18 (40.9%)	2 (4.5%)	9 (20.5%)	0 (0.0%)	9 (20.5%)	3 (6,8%)	3 (6.8%)
	(+)	16 (51.6%)	1 (3.2%)	5 (16.1%)	1 (3.2%)	4 (12.9%)	2 (6.5%)	2 (6.5%)
CRVO	(-)	9 (50.0%)	1 (5.6%)	0 (0.0%)	1 (5.6%)	2 (11.1%)	3 (16.7%)	2 (11.1%)
	(+)	11 (45.8%)	3 (12.5%)	0 (0.0%)	1 (4.2%)	5 (20.8%)	3 (12.5%)	1 (4.2%)

BRVO= branch retinal vein occlusion; CRVO= central retinal vein occlusion; Dexa. İmp.= intravitreal sustained-release dexamethasone implant; RVO= retinal vein occlusion; SMD= serous macular detachment.

The baseline and final visit mean VA values in BRVO eyes were 0.86 ± 0.46 and 0.63 ± 0.49 logMAR (p<0.001), respectively, in SMD (-) cases and 0.87 ± 0.46 and 0.66 ± 0.53 logMAR (p=0.054), respectively, in SMD (+) cases. The baseline and final visit mean VA values in the CRVO group were 1.51 ± 0.59 and 1.20 ± 0.54 logMAR (p=0.029), respectively, in SMD (-) cases and 1.03 ± 0.71 and 1.06 ± 0.71 logMAR (p=0.791), respectively, in SMD (+) cases. No significant difference was found in the mean VA between SMD (-) and SMD (+) eyes with BRVO or CRVO at any time point. A significant increase was detected in the mean VA compared to the baseline value at all visits in SMD (-) eyes with BRVO. However, the mean VA change compared to the baseline value showed a significant increase only at month 3 in SMD (+) eyes with BRVO. In SMD (-) eyes with CRVO, the mean VA increased at all visits but demonstrated a statistical significance only at month 6 compared to the baseline value. However, in SMD (+) eyes with CRVO, there was an increase that was not statistically significant only at month 3 and a decrease at other visits in the mean VA compared to the baseline value (Table 3).

**Table 3:** The course of VA according to the SMD status in eyes with RVO and the statistical analysis compared to the baseline

		Initial VA (logMAR)	Month 3 VA (logMAR)	Month 6 VA (logMAR)	Month 9 VA (logMAR)	Month 12 VA (logMAR)	p value
BRVO	SMD (-)	0.86 ± 0.46 (n=44, 100%)	0.71 ± 0.52 (n=34, 77.3%)	0.66 ± 0.50 (n=33, 75.0%)	0.77 ± 0.48 (n=26, 59.1%)	0.74 ± 0.54 (n=21,47.7%)	p_1_: 0.005^*^ p_2_: 0.002^*^ p_3_: 0.012^*^ p_4_: 0.035^*^
	SMD (+)	0.87 ± 0.46 (n=31, 100%)	0.67 ± 0.57 (n=23, 74.2%)	0.63 ± 0.46 (n=21, 67.7%)	0.79 ± 0.48 (n=19, 61.3%)	0.93 ± 0.52 (n=16, 51.6%)	p_1_: 0.04^*^ p_2_: 0.058 p_3_: 0.194 p_4_: 0.470
	P_0_	0.944	0.590	0.865	0.744	0.292	
CRVO	SMD (-)	1.51 ±0.59(n=18, 100%)	1.13 ± 0.54 (n=8, 44,4%)	1.13 ± 0.87 (n=10, 55.5%)	1.21 ± 0.51 (n=12, 66.7%)	1.19 ± 0.49 (n=10, 55.5%)	p_1_: 0.095 p_2_: 0.045^*^ p_3_: 0.078 p_4_: 0.176
	SMD (+)	1.03 ± 0.71 (n=24, 100%)	0.63 ± 0.51 (n = 11,45.8%)	1.09 ± 0.55 (n=14, 58.3%)	1.32 ± 0.59 (n=10, 41.6%)	1.03 ± 0.55 (n=10,41.6%)	p_1_: 0.455 p_2_: 0.554 p_3_: 0.313 p_4_: 0.204
	P_0_	0.009^*^	0.056	0.905	0.645	0.589	

BRVO= branch retinal vein occlusion; CRVO= central retinal vein occlusion; RVO= retinal vein occlusion; SMD= serous macular detachment; VA= visual acuity. p_0_= Statistical comparison of VA between those with and without SMD at each follow-up. p_1_= Statistical analysis result of change at month 3 compared to baseline. p_2_= Statistical analysis result of change at month 6 compared to baseline. p_3_= Statistical analysis result of change at month 9 compared to baseline. p_4_= Statistical analysis result of change at month 12 compared to baseline.

* = Statistically significant difference.

In the absence of macular ischemia, in the SMD (-) group (n=50), the mean initial and final VA values were 0.96 ± 0.53 and 0.66 ± 0.51 logMAR (p<0.001), respectively, and in the SMD (+) group (n=43), the respective values were 0.79 ± 0.44 and 0.64 ± 0.48 logMAR (p=0.056). In the presence of macular ischemia, in the SMD (-) group (n=12), the mean initial and final VA values were 1.43 ± 0.66 and 1.38 ± 0.37 logMAR (p=0.498), respectively, and in the SMD (+) group (n=12), the respective values were 1.45 ± 0.72 and 1.51 ± 0.71 logMAR (p=0.678). Ellipsoid zone damage was significantly more prominent in SMD (+) eyes than in SMD (-) eyes in the BRVO group (p=0.032). It was also more prominent in SMD (+) eyes than in SMD (-) eyes in the CRVO group, but there was no statistically significant difference (p=0.276). There was also no statistically significant difference between ELM damage rates in SMD (-) and SMD (+) cases in either the BRVO or CRVO group. Only in the absence of macular ischemia did the ellipsoid zone damage increase significantly in the SMD (+) group (p=0.003). In the presence and absence of macular ischemia, the ELM damage was not affected by the presence of SMD (p=0.105) (Table 4). In eyes without ELM damage in the SMD (-) (n=43) and SMD (+) (n=33) groups, the mean final VA values were 0.54 ± 0.44 and 0.49 ± 0.42 logMAR, respectively (p=0.651). In eyes with ELM damage in the SMD (-) (n=19) and SMD (+) (n=22) groups, the mean final VA values were 1.37 ± 0.35 and 1.33 ± 0.58 logMAR, respectively (p=0.747). In the group with ELM damage, the mean VA at baseline and at the final visit was significantly higher than that in the group without ELM damage (p<0.001 for both). SMD recurred once in two eyes (6.4%) and twice in one eye (3.2%) after resolution in the BRVO group. Similarly, SMD recurred once in two eyes (8.3%) and twice in three eyes (12.5%) after resolution in the CRVO group. In the BRVO group, the final VA values were 0.30, 0.30, and 1.51 logMAR, and the CFT values were 541, 239, and 220 µm in eyes with relapsed SMD, respectively. In eyes with relapsed SMD in the CRVO group, the mean final VA was 1.04 (0.501.30) logMAR, and the mean final CFT was 354.6 µm (55-670). There was no later SMD development in any of the eyes without initial SMD. None of the eyes had SMD at the final visit.

**Table 4:** Distribution of the ellipsoid zone and ELM damage according to the RVO type, macular ischemia, and SMD presence

		Ellipsoid zone damage		ELM damage	
**RVO Type**		(-)	(+)	(-)	(+)
BRVO	SMD (-)	32 (72.7%)	12 (27.3%)	33 (75.0%)	11 (25.0%)
	SMD (+)	15(48.4%)	16(51.6%)	21(67.7%)	10(32.3%)
	P	p=0.032^*^		p=0.491	
CRVO	SMD (-)	7(38.9%)	11(61.1%)	10(55.6%)	8(44.4%)
	SMD (+)	10 (41.7%)	14 (58.3%)	12 (50.0%)	12 (50.0%)
	P	p=0.276		p=0.721	
Macular ischemia (-)	SMD (-)	40(80.0%)	10(20.0%)	41(82.0%)	9(18.0%)
	SMD (+)	22(51.2%)	21(48.8%)	29(67.4%)	14(32.6%)
	P	p = 0.003^*^		p=0.105	
Macular ischemia (+)	SMD (-)	1 (8.3%)	11 (91.7%)	2 (16.7%)	10 (83.3%)
	SMD (+)	1 (8.3%)	11 (91.7%)	4 (33.3%)	8 (66.7%)
	p value	p= 1.000		p=0.640	

BRVO= branch retinal vein occlusion; CRVO= central retinal vein occlusion; RVO= retinal vein occlusion; SMD= serous macular detachment; ELM= external limiting membrane.

* = Statistically significant difference.

In the BRVO group, the baseline and final visit mean CFT values were 542.30 ± 182.12 and 270.20 ± 99.42 µm (p<0.001), respectively, in SMD (-) cases and 546.4 ± 159.35 and 281 ± 118.41 µm (p<0.001), respectively, in SMD (+) cases. In the CRVO group, the baseline and final visit mean CFT values were 707.00 ± 201.77 and 308.38 ± 116.29 µm (p<0.001), respectively, in SMD (-) cases and 645.29 ± 240.63 and 264.00 ± 120.94 µm (p<0.001), respectively, in SMD (+) cases. No statistically significant difference was found in the mean CFT values between SMD (-) and SMD (+) eyes at any time point in the BRVO and CRVO groups. The mean CFT showed a decrease compared to the baseline value at all time points in both SMD (-) and SMD (+) eyes with BRVO or CRVO (Table 5).

**Table 5:** The course of CFT according to the SMD status in eyes with RVO and the statistical analysis compared to the baseline

	Initial CFT (µm)	Month 3 CFT (µm)	Month 6 CFT (µm)	Month 9 CFT (µm)	Month 12 CFT (µm)	p value
BRVO SMD (-)	542.3±182.1 (n=44, 100%)	321.9±153.3 (n=34, 77.2%)	387.8±176.7 (n=34, 77.2%)	322.0±157.0 (n=27, 61.3%)	352.4±164.5 (n=25, 56.8%)	p_1_ <0.001^*^ p_2_<0.001^*^ p_3_<0.001^*^ p_4_<0.001^*^
SMD (+) P_0_	546.4±159.4 (n=31, 100%) 0.906	301.3±121.51 (n=24, 77.4%) 0.758	401.9± 176.5 (n=24, 77.4%) 0.766	325.9±137.2 (n=20, 64.5%) 0.914	303.9±169.8 (n=17, 54.8%) 0.155	p_1_ <0.001^*^ p_2_<0.001^*^ p_3_<0.001^*^ p_4_<0.001^*^
CRVO SMD (-)	707.0±201.8 (n=18, 100%)	437.4±215.1 (n=9, 50.0%)	507.1 ±277.5 (n=10, 55.5%)	431.9±164.3 (n=14, 77.7%)	396.1 ±195.2 (n=9, 50.0%)	p_1_: 0.005^*^ p_2_: 0.137 p_3_: 0.003^*^ p_4_: 0.00T^*^
SMD (+) P_0_	645.3±240.6 (n=24, 100%) 0.384	351.8±201.2 (n=12, 50.0%) 0.360	340.4±225.8 (n=14, 58.3%) 0.079	512.0±275.9 (n=10,41.6%) 0.382	468.5±232.7 (n=11,45.8%) 0.468	p_1_: 0.003^*^ p_2_: 0.008^*^ p_3_: 0.024^*^ p_4_: 0.110

BRVO= branch retinal vein occlusion; CRVO= central retinal vein occlusion; RVO= retinal vein occlusion; SMD= serous macular detachment; VA= visual acuity

p_0_= Statistical comparison of VA between those with and without SMD at each follow-up. p_1_= Statistical analysis result of change at month 3 compared to baseline. p_2_= Statistical analysis result of change at month 6 compared to baseline. p_3_= Statistical analysis result of change at month 9 compared to baseline. p_4_= Statistical analysis result of change at month 12 compared to baseline.

* = Statistically significant difference.

## DISCUSSION

The mechanism underlying SMD development is still not completely clear. The possible causes of subretinal fluid collection are vascular leakage from the retinal and/or choroidal circulation and disturbed fluid removal function of the retinal pigment epithelium secondary to increased inflammation^(10,11)^. The fact that both macular edema and SMD respond to anti-inflammatory treatment confirms the effect of inflammation in RVO on the pathogenesis of both conditions. Some studies have reported that increased VEGF levels in the vitreous and aqueous humor support the role of increased inflammation in SMD development^(12,13)^.

Inflammation plays a significant role in SMD development; however, the fact that SMD is not observed in every RVO case indicates that inflammation is not the only factor in the mechanism underlying SMD development. Therefore, the effect of inflammation on SMD development can be explained by changes in the natural barriers and the microstructure^(7)^.

The possible involvement of the outer blood-retina barrier during SMD development indicates a pathological spread of the process of RVO from inner retinal layers to outer retinal layers. The inflammatory mediators that pass toward outer retinal layers could play a role in this spread. The ELM consists of cells in the interface between the intercellular connections of the inner segment of photoreceptors and Müller cells^(14)^, and its pores are not permeable to large serum proteins such as albumin and globulins^(15,16)^. It is possible that inflammatory molecules of a size that can pass through the ELM pores can also pass through the intercellular space^(17)^, and the outer blood-retina barrier could be broken down due to the effect of these molecules^(14,18,19)^. Therefore, proteins can collect in the subretinal area^(20,21)^ and draw water by crea ting a hyperosmotic section compared to that in inner retinal layers. Moreover, the decreased water removal capacity of the RPE due to inflammation can increase the collection of fluid beyond capacity and result in SMD development by opening up the potential space within the subretinal region. However, ELM defects due to inflammation can develop at later periods and prevent the function of the ELM osmotic barrier. Proteins can easily pass through the ELM defects when the ELM loses its protein barrier function, thereby preventing the development of an osmotic gradient between the two sides of the ELM^(14)^. This thesis is supported by the facts that there were only a few SMD relapses despite severe macular edema relapses and no case with SMD development without RVO initiation in this study. Early ELM damage and inflammation that does not cause adequate disruption of the other blood-retina barrier could explain the other cases in which no SMD development was observed.

SMD is a complication reported at a rate of 28.3%71.4% in BRVO cases and 50.0%-100.0% in CRVO cases; SMD has been evaluated as a factor that could influence treatment outcomes^(5,8,22-24)^. Similarly, we found the rate of SMD to be 41.3% in eyes with BRVO and 57.1% in those with CRVO. Considering the higher inflammation and vascular leakage in eyes with CRVO than in eyes with BRVO^(25)^, the higher rate of SMD development in eyes with CRVO could provide some proof that increased inflammation and vascular leakage are associated with SMD development.

Although some studies associate the presence of SMD in RVO with a poor visual prognosis, the effect of SMD on visual prognosis still remains unclear^(4,6,18,22,26,27)^. The evaluation of our patients after dividing them into BRVO and CRVO groups revealed that the early gain in mean VA in the SMD (+) group was either lost or became insignificant over time in both patients with CRVO and those with BRVO. Furthermore, there was a meaningful increase in the mean VA value at the final visit in SMD (-) BRVO eyes but not in SMD (+) eyes. The mean CFT decreased at all time points in both SMD (+) and SMD (-) eyes with BRVO or CRVO. This result indicates that the final visual prognosis was worse in the SMD (+) group despite the similar anatomic improvement in the two groups. The increase in VA in the early period could have been obtained as a result of the recovery of contact between the neurosensorial retina and RPE following successful SMD treatment. However, this increase in VA could be lost over time because of the larger quantity of inflammatory factors^(12,13)^, the mechanical photoreceptor damage that occurs during SMD development, and the gradual increase in metabolic photoreceptor damage as a result of the loss of contact between photoreceptors and RPE due to SMD^(7,22,28)^. The absence of a significant difference between the SMD groups with regard to the final CFT result has similarly been reported in several studies and supports our findings^(4,13,18,22)^.

Our results demonstrate that the therapeutic potential of IV treatments used for macular edema of RVO origin also applies to SMD treatment. Although there were patients with SMD recurrence, all recurrences had been resolved by the final visit. Nevertheless, the low number of patients with SMD recurrence prevents us from reaching a conclusion regarding the prognosis. The present medical treatments cannot improve or recover the photoreceptor damage that develops before treatment but can decrease vascular damage and leakage and therefore treat SMD and macular edema with every administration. This could explain the differentiation of CFT and VA results in the SMD (+) group over time.

The negative effect of SMD on visual prognosis is also supported by the OCT findings. Tsujikawa et al. reported a significantly more prominent IS/OS defect in the SMD (+) group, but they did not find a significant difference in ELM defects between the SMD (-) and SMD (+) groups^(7)^. Similarly, we found more prominent ellipsoid zone damage in SMD (+) BRVO eyes. In contrast, ellipsoid zone damage was similarly distributed in SMD (-) and SMD (+) cases in the CRVO group. We believe that this could be the result of the much larger ellipsoid zone damage potential from CRVO itself rather than from SMD. Consequently, the ellipsoid zone damage of SMD may be masked by CRVO. Similarly, due to the masking of macular ischemia, the negative effect of SMD on the ellipsoid zone becomes evident only in the absence of macular ischemia. Our microstructural findings are supported by similar findings in the course of VA.

We found no significant difference in ELM damage based on the presence of SMD irrespective of the RVO type and macular ischemia status. This result may indicate that ELM damage is more a part of the RVO process than a harmful effect of SMD. When eyes with and without ELM damage were examined in two separate groups, the mean VA of those with ELM damage was lower at baseline and at the final visit. However, no significant difference was detected in the mean VA based on the presence of SMD in each group. These results indicate that ELM damage is associated with poor visual prognosis from the beginning and that ELM status is more a determinant of VA than SMD status. The limitations of our study include its retrospective nature, the wide range of options for the treatment regimen, the possibility that the result was influenced by the chosen treatment, and the small sample size for the evaluation of some subgroups.

In conclusion, we detected a negative effect of the presence of SMD on VA in eyes with BRVO or CRVO. We believe that the reason for this finding is the additional photoreceptor damage due to inflammatory, mechanical, or metabolic causes in SMD (+) eyes. Moreover, it is possible that ELM integrity plays a significant role in the development and recurrence of SMD. Nonetheless, further randomized controlled studies on larger series are required to clarify these results.

## References

[1] Hayreh SS. (2005). Prevalent misconceptions about acute retinal vascular occlusive
disorders. Prog Retin Eye Res.

[2] The Branch Vein Occlusion Study Group (1984). Argon laser photocoagulation for macular edema in branch vein
occlusion. Am J Ophthalmol.

[3] Chung EJ, Hong YT, Lee SC, Kwon OW, Koh HJ. (2008). Prognostic factors for visual outcome after intravitreal
bevacizumab for macular edema due to branch retinal vein
occlusion. Graefes Arch Clin Exp Ophthalmol.

[4] Hoeh AE, Ruppenstein M, Ach T, Dithmar S. (2010). OCT patterns of macular edema and response to bevacizumab therapy
in retinal vein occlusion. Graefes Arch Clin Exp Ophthalmol.

[5] Jaissle GB, Szurman P, Feltgen N, Spitzer B, Pielen A, Rehak M, Retinal Vein Occlusion Study Group (2011). Predictive factors for functional improvement after intravitreal
bevacizumab therapy for macular edema due to branch retinal vein
occlusion. Graefes Arch Clin Exp Ophthalmol.

[6] Poon YC, Chen CH, Kuo HK, Chen YJ, Wu PC, Chen YH (2013). Clinical implications of serous retinal detachment in branch
retinal vein occlusion and response after primary intravitreal bevacizumab
injection. J Ocul Pharmacol Ther.

[7] Tsujikawa A, Sakamoto A, Ota M, Kotera Y, Oh H, Miyamoto K (2010). Serous retinal detachment associated with retinal vein
occlusion. Am J Ophthalmol.

[8] Dolz-Marco R, Gallego-Pinazo R, Sanz-Marco E, Díaz-Llopis M, Lleó-Pérez A. (2011). [Influence of serous macular detachment on the efficacy of
ranibizumab treatment in retinal vein occlusions]. Arch Soc Esp Oftalmol.

[9] Balci Ö, Öngör E. (2004). Retinal Vein Occlusions. Turk J Ophthalmol.

[10] Catier A, Tadayoni R, Paques M, Erginay A, Haouchine B, Gaudric A (2005). Characterization of macular edema from various etiologies by
optical coherence tomography. Am J Ophthalmol.

[11] Marmor MF. (1999). Mechanisms of fluid accumulation in retinal edema. Doc Ophthalmol.

[12] Noma H, Funatsu H, Mimura T, Tatsugawa M, Shimada K, Eguchi S. (2012). Vitreous inflammatory factors and serous macular detachment in
branch retinal vein occlusion. Retina.

[13] Park SP, Ahn JK, Mun GH. (2010). Aqueous vascular endothelial growth factor levels are associated
with serous macular detachment secondary to branch retinal vein
occlusion. Retina.

[14] Daruich A, Matet A, Moulin A, Kowalczuk L, Nicolas M, Sellam A (2018). Mechanisms of macular edema: beyond the surface. Prog Retin Eye Res.

[15] Bunt-Milam AH, Saari JC, Klock IB, Garwin GG. (1985). Zonulae adherentes pore size in the external limiting membrane of
the rabbit retina. Invest Ophthalmol Vis Sci.

[16] Omri S, Omri B, Savoldelli M, Jonet L, Thillaye-Goldenberg B, Thuret G (2010). The outer limiting membrane (OLM) revisited: clinical
implications. Clin Ophthalmol.

[17] Dib E, Maia M, Longo-Maugeri IM, Martins MC, Mussalem JS, Squaiella CC (2008). Subretinal bevacizumab detection after intravitreous injection in
rabbits. Invest Ophthalmol Vis Sci.

[18] Gallego-Pinazo R, Dolz-Marco R, Pardo-López D, Martínez-Castillo S, Lleó-Pérez A, Arévalo JF (2013). Ranibizumab for serous macular detachment in branch retinal vein
occlusions. Graefes Arch Clin Exp Ophthalmol.

[19] Omri S, Behar-Cohen F, Rothschild PR, Gélizé E, Jonet L, Jeanny JC (2013). PKCζ mediates breakdown of outer blood-retinal barriers in
diabetic retinopathy. PLoS One.

[20] Marmor MF. (1985). Barriers to fluorescein and protein movement. Jpn J Ophthalmol.

[21] Marmor MF. (1990). Control of subretinal fluid: experimental and clinical
studies. Eye (Lond).

[22] Maggio E, Polito A, Guerriero M, Pertile G. (2014). Intravitreal dexamethasone implant for macular edema secondary to
retinal vein occlusion: 12-month follow-up and prognostic
factors. Ophthalmologica.

[23] Noma H, Funatsu H, Mimura T, Shimada K. (2011). Visual function and serous retinal detachment in patients with
branch retinal vein occlusion and macular edema: a case
series. BMC Ophthalmol.

[24] Spaide RF, Lee JK, Klancnik Jr, JK (2003). Gross NE. Optical coherence tomography of branch retinal vein
occlusion. Retina.

[25] Deobhakta A, Chang LK. (2013). Inflammation in retinal vein occlusion. Int J Inflamm.

[26] Shroff D, Mehta DK, Arora R, Narula R, Chauhan D. (2008). Natural history of macular status in recent-onset branch retinal
vein occlusion: an optical coherence tomography study. Int Ophthalmol.

[27] Celık E, Doğan E, Turkoglu EB, Çakır B, Alagoz G. (2016). Serous retinal detachment in patients with macular edema
secondary to branch retinal vein occlusion. Arq Bras Oftalmol.

[28] Ohashi H, Oh H, Nishiwaki H, Nonaka A, Takagi H. (2004). Delayed absorption of macular edema accompanying serous retinal
detachment after grid laser treatment in patients with branch retinal vein
occlusion. Ophthalmology.

